# Supplementary material for: Anti-LRP5/6 VHHs promote differentiation of Wnt-hypersensitive intestinal stem cells
Source: Nat Commun. 2019 Jan 21;10:365. doi: 10.1038/s41467-018-08172-z (PMC6341108; doi:10.1038/s41467-018-08172-z)
Supplement: Supplementary file 1 — Supplementary Information [file 41467_2018_8172_MOESM1_ESM.pdf]

**Supplementary information**

**Anti-LRP5/6 VHHs promote differentiation of Wnt-hypersensitive intestinal stem cells**

**N. Fenderico, R. C van Scherpenzeel, M. Goldflam et al.**

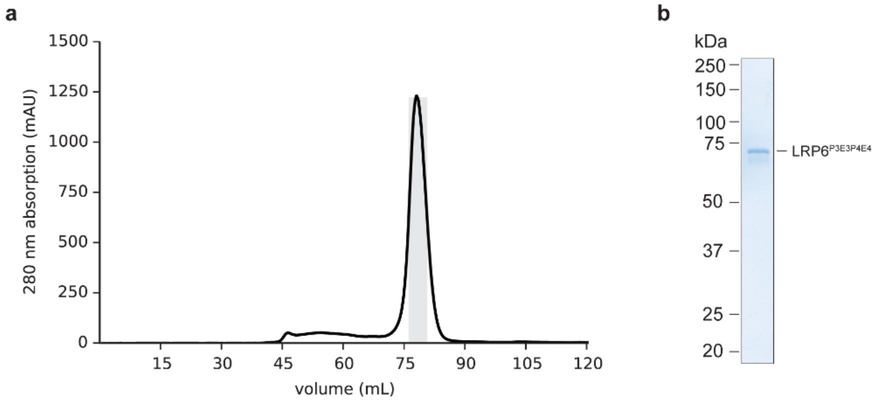


**Supplementary Figure 1. Purification of human LRP6$^{P3E3P4E4}$. a)** Purified C-terminal $His_6$-tagged LRP6 was separated by SEC on a Superdex 200 16/60 column, showing a monodispersed peak. **b)** Reducing SDS-PAGE of the peak fraction used for X-ray crystallography studies showed a single 70 kDa band.

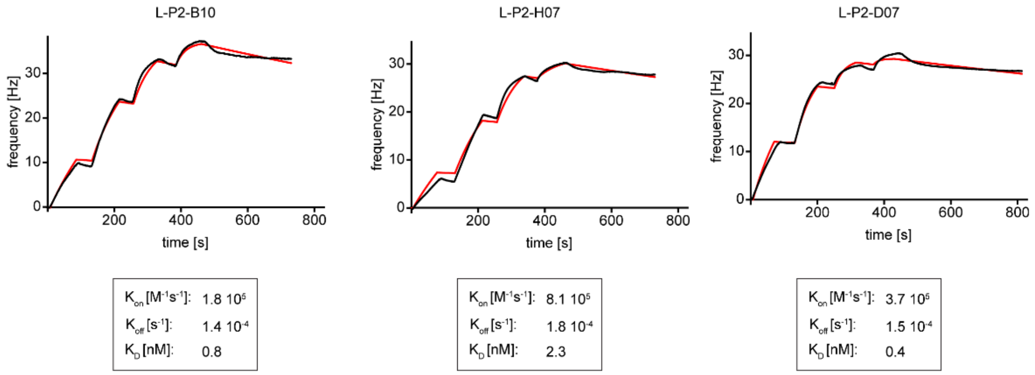


**Supplementary Figure 2. QCM kinetic interaction determination of VHH-LRP6$^{P3E3P4E4}$ complexes.** Sensorgrams showing binding between anti-LRP6 VHH and immobilized LRP6$^{P3E3P4E4}$ measured in Hertz as a function of time by a single cycle kinetic assay using increasing concentrations of VHH ranging from 30 pM to 250 pM. The black lines represent data and the red lines represent fitted curves.

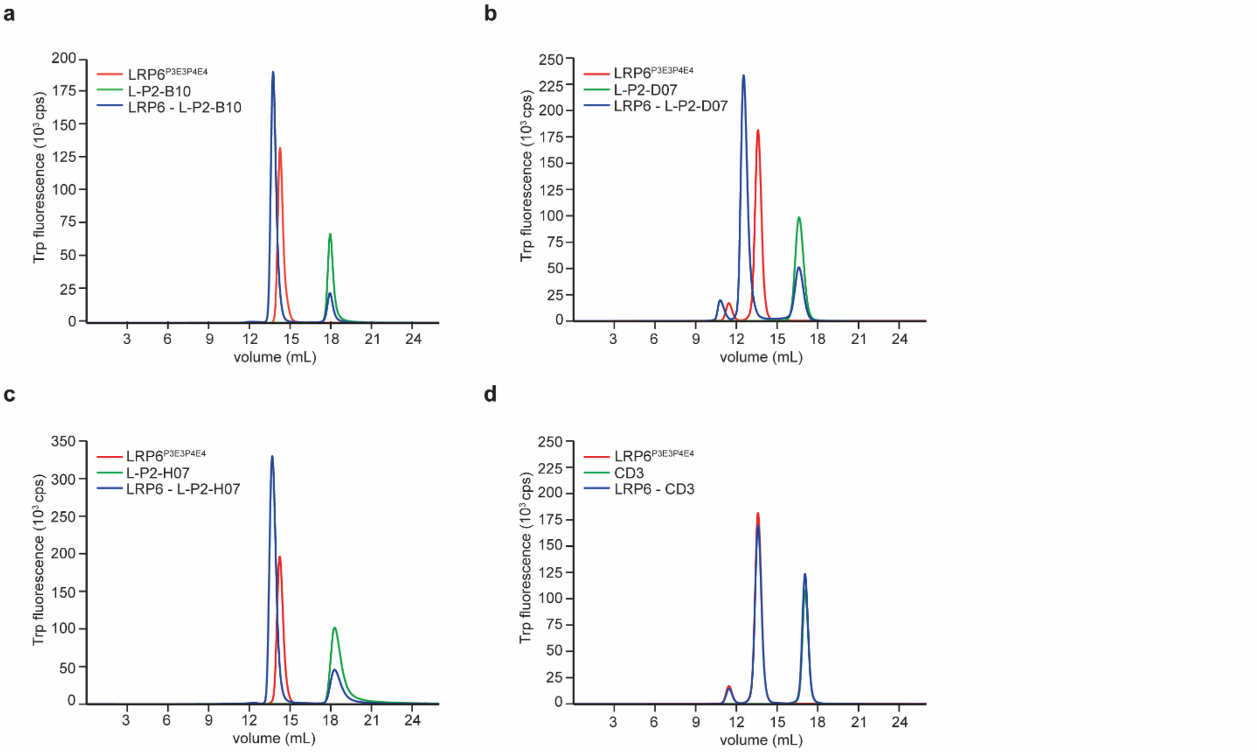


**Supplementary Figure 3. Size-exclusion chromatograms of LRP6$^{P3E3P4E4}$, VHH and LRP6$^{P3E3P4E4}$-VHH. a-c)** Complex formation of LRP6$^{P3E3P4E4}$ with **(a)** L-P2-B10-His$_6$ tagged, **(b)** L-P2-D07-V5-Flag$_3$-His$_6$ tagged and **(c)** L-P2-H07-His$_6$ tagged. A peak-shift is observed upon binding with the VHH. Note that a slightly bigger peak-shift is observed for L-P2-D07-V5-Flag$_3$-His$_6$, due to the larger tag on the VHH. **d)** Negative control with VHH-CD3. No peak shift is observed when incubating VHH-CD3 with LRP6$^{P3E3P4E4}$.

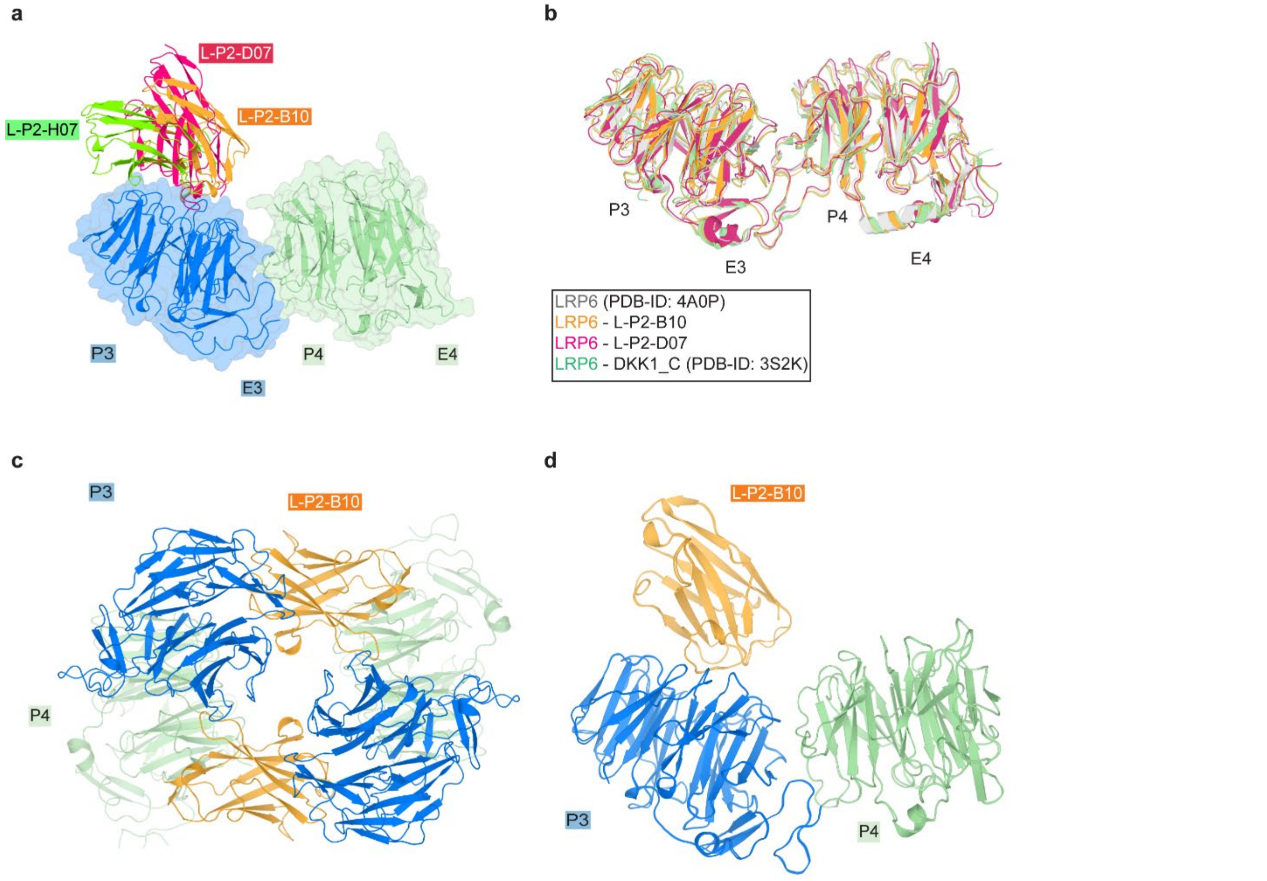


**Supplementary Figure 4. Structural analysis of $LRP6^{P3E3P4E4}$ with VHHs L-P2-B10, L-P2-D07 and L-P2-H07. a)** Cartoon representation of three $LRP6^{P3E3P4E4}$ - VHHs structures superposed based on the $\beta$-propeller domains of LRP6. One LRP6 molecule is presented with P3E3 indicated in blue and P4E4 in green. The VHH L-P2-B10 is indicated in orange, L-P2-D07 in magenta and L-P2-H07 in green. **b)** Cartoon representation of $LRP6^{P3E3P4E4}$ (LRP6-L-P2-B10 in orange, LRP6-L-P2-D07 in magenta, LRP6 (PDB-ID: 4A0P) in grey and LRP6-DKK1_C (PDB-ID: 3S2K) in green superposed reflecting minor domain reorientations in the EGF regions. One loop comprising residues 1005-1012 in $\beta$-propeller domain 4 was not visible in the electron density map of either crystal structures. **c)** $LRP6^{P3E3P4E4}$-L-P2-B10 packing with two complexes in the asymmetric unit (ASU). **d)** The two complexes superposed well with an rmsd of 0.2 Å (for 665 out of 733 $C\alpha$-atoms). The contacts stabilizing the interaction between the VHH and $LRP6^{P3}$ domain are identical in the two copies.

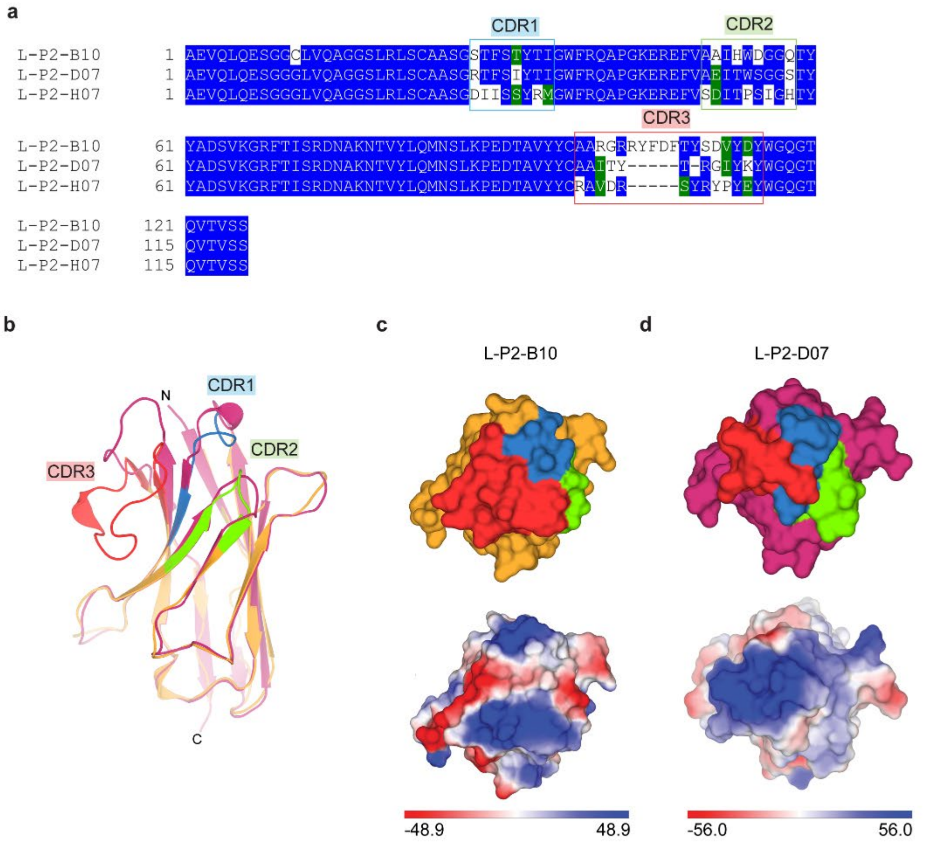


**Supplementary Figure 5. Structural analysis of VHHs L-P2-B10 and L-P2-D07. a)** Identical amino acids are highlighted in blue. Homologous amino acids are highlighted in green. Residues forming the CDR region are indicated with boxes (CDR1 in blue, CDR2 in green and CDR3 in red). **b)** Superposition of the two VHH domain crystal structures from the LRP6-L-P2-B10 and LRP6-L-P2-D07 complexes (color scheme as in Figure 3). CDR regions are indicated for LRP6-L-P2-B10 in the color scheme of **(a)**. **c-d)** Surface representation of VHH L-P2-B10 **(c)** and VHH L-P2-D07 **(d)** viewed from the CDR paratope region of the molecules. Electrostatics surface representation of the same view showing the CDR region on both VHHs in bottom panels.

```
LRP6P3  631 EAFLLFSRRADIRRISLETNNNNVAIPLTGVKEASALDFDVTDNRIYWTDISLKTISRAF
LRP5P3  644 EAFLVFTSRAAIHRISLETNNNDVAIPLTGVKEASALDFDVSNNHIYWTDVSLKTISRAF

LRP6P3  689 MNGSALEHVVEFGLDYPEGMAVDWLGKNLYWADTGTNRIEVSKLDGQHRQVLVWKDLDSP
LRP5P3  702 MNGSSVEHVVEFGLDYPEGMAVDWMGKNLYWADTGTNRIEVARLDGQFRQVLVWRDLDNP

LRP6P3  738 RALALDPAEGFMYWTEWGGKPKIDRAAMDGSERTTLVPNVGRANGLTIDYAKRRLYWTDL
LRP5P3  751 RSLALDPTKGYIYWTEWGGKPRIVRAFMDGTNCMTLVDKVGRANDLTIDYADQRLYWTDL

LRP6P3  796 DTNLIESSNMLGLNREVIADDLPHPFGLTQYQDYIYWTDWSRRSIERANKTSGQNRTIIQ
LRP5P3  809 DTNMIESSNMLGQERVVIADDLPHPFGLTQYSDYIYWTDWNLHSIERADKTSGRNRTLIQ

LRP6P3  855 GHLDYVMDILVFHSSRQSGWNECASSNGHCSHLCLAVPVGGFVCGCPAHYSLNADNRTCS
LRP5P3  868 GHLDFVMDILVFHSSRQDGLNDCMHNNGQCGQLCLAIP-GGHRCGCASHYTLDPSSRNCS

LRP6P3  912 AP
LRP5P3  924 PP
```

**Supplementary Figure 6. High conservation of VHH contact residues between human $LRP5^{P3}$ and $LRP6^{P3}$.** Sequence alignment of the P3 domain of human LRP5 and LRP6. Similar (blue) and identical (red) residues are indicated. Yellow boxes indicate residues previously determined to interact with DKK1; black dots indicate residues that interact with VHH, as determined in Figure 3.

*LRP6 N117Fs*

```
L  A  C  D  W  L  G  E  K  L  Y  W  T  D  S  E  T   N  R  I  E  V  S  N  L  D
CTGGCATGTGATTGGCTTGGAGAAAAATTGTACTGGACAGATTCTGAAACT-AATCGGATTGAAGTTTCTAATTTAG
||||||||||||||||||||||||||||||||||||||||||||||||||| |||||||||||||||||||||||||
CTGGCATGTGATTGGCTTGGAGAAAAATTGTACTGGACAGATTCTGAAACTTAATCGGATTGAAGTTTCTAATTTAG
L  A  C  D  W  L  G  E  K  L  Y  W  T  D  S  E  T  *  S  D  *  S  F  *  F  R
```

*LRP6 N117Fs*

```
L  A  C  D  W  L  G  E  K  L  Y  W  T  D  S  E  T    N  R  I  E  V  S  N  L  D
CTGGCATGTGATTGGCTTGGAGAAAAATTGTACTGGACAGATTCTGAAACT--AATCGGATTGAAGTTTCTAATTTAG
|||||||||||||||||||||||||||||||||||||||||||||||||||  |||||||||||||||||||||||||
CTGGCATGTGATTGGCTTGGAGAAAAATTGTACTGGACAGATTCTGAAACTGAAATCGGATTGAAGTTTCTAATTTAG
L  A  C  D  W  L  G  E  K  L  Y  W  T  D  S  E  T  E  I  G  L  K  F  L  I  *
```

**Supplementary Figure 7. CRISPR-Cas9-based strategy for *LRP6* knock out cell line.** Disruption of *LRP6* was achieved by a CRISPR-Cas9-based strategy. The two knock out alleles are represented together with the sequence of wild type LRP6 (bottom strand). The gRNA sequence is highlighted in red.

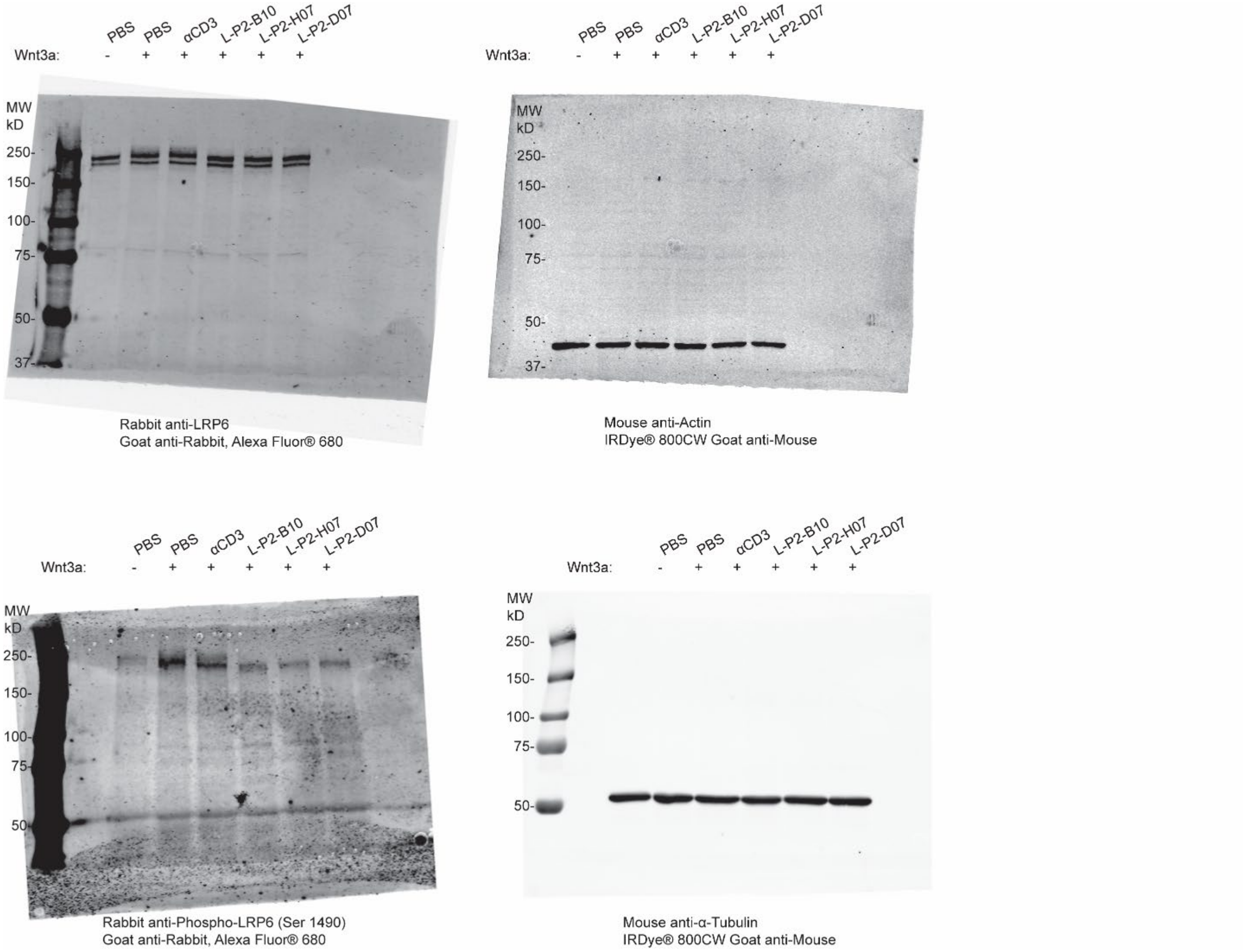


**Supplementary Figure 8. Full blots related to Figure 4e.**

**Supplementary Table 1: primers used for generating *LRP6* knockout cell line**

| | Fw | Rev |
|---|---|---|
| *LRP6* gRNA | 5'-CACCGGGACAGATTCTGAAACTAAT-3' | 5'-AAACATTAGTTTCAGAATCTGTCCC-3' |
| *LRP6* genotyping | 5'-CTTTATGCAAACAGACGGGACT-3' | 5'-AAAGCAAAAACCCTGTCAAAAA-3' |

**Supplementary Table 2: qRT-PCR primers used in this study**

| Gene | Fw | Rev |
|---|---|---|
| *Hprt* | 5'-AAGCTTGCTGGTGAAAAGGA-3' | 5'-TTGCGCTCATCTTAGGCTTT-3' |
| *Lgr5* | 5'-AGAACACTGACTTTGAATGG-3' | 5'-CACTTGGAGATTAGGTAACTG-3' |
| *Olfm4* | 5'-GCCACTTTCCAATTTCAC-3' | 5'-GAGCCTCTTCTCATACAC-3' |
| *Axin2* | 5'-GGACTGGGGAGCCTAAAGGT-3' | 5'-AAGGAGGGACTCCATCTACGC-3' |
| *Lys1* | 5'-GGAATGGATGGCTACCGTGG-3' | 5'-CATGCCACCCATGCTCGAAT-3' |
| *ChgA* | 5'-CGATCCAGAAAGATGATGGTC-3' | 5'-CGGAAGCCTCTGTCTTTCC-3' |
| *Muc2* | 5'-GCTGACGAGTGGTTGGTGAATG-3' | 5'-GATGAGGTGGCAGACAGGAGAC-3' |
| *Alpi* | 5'-GGCTACACACTTAGGGGGACCTCCA-3' | 5'-AGCTTCGGTGACATTGGGCCGGT-3' |

**Supplementary Table 3: Cis display selection and ELISA screening primers**

| | Fw | Rev |
|---|---|---|
| *RepA* | 5'-AATCTCGGAAGGACGCTTCA-3' | 5'-TTTTGCGCTTCACCTCGC-3' |
| *TAC6* | 5'-CCCCATCCCCCTGTTGACAATTAATC-3' | |
| *NOTIRECREV* | 5'-TGGTGAAGATCAGTTGCGGCCGCTAG-3' | |
