# Supplementary material for: Anti-LRP5/6 VHHs promote differentiation of Wnt-hypersensitive intestinal stem cells
Source: Nat Commun. 2019 Jan 21;10:365. doi: 10.1038/s41467-018-08172-z (PMC6341108; doi:10.1038/s41467-018-08172-z)
Supplement: Supplementary file 3 — Reporting Summary [file 41467_2018_8172_MOESM3_ESM.pdf]

nature research

Corresponding author(s): *Double-blind peer review submissions: write DBPR and your manuscript number here instead of author names.*

Reporting Summary

Nature Research wishes to improve the reproducibility of the work that we publish. This form provides structure for consistency and transparency in reporting. For further information on Nature Research policies, see Authors & Referees and the Editorial Policy Checklist.

Statistical parameters

When statistical analyses are reported, confirm that the following items are present in the relevant location (e.g. figure legend, table legend, main text, or Methods section).

n/a	Confirmed	
☐	☒	The exact sample size (*n*) for each experimental group/condition, given as a discrete number and unit of measurement
☐	☒	An indication of whether measurements were taken from distinct samples or whether the same sample was measured repeatedly
☒	☐	The statistical test(s) used AND whether they are one- or two-sided<br>*Only common tests should be described solely by name; describe more complex techniques in the Methods section.*
☒	☐	A description of all covariates tested
☒	☐	A description of any assumptions or corrections, such as tests of normality and adjustment for multiple comparisons
☐	☒	A full description of the statistics including central tendency (e.g. means) or other basic estimates (e.g. regression coefficient) AND variation (e.g. standard deviation) or associated estimates of uncertainty (e.g. confidence intervals)
☒	☐	For null hypothesis testing, the test statistic (e.g. *F*, *t*, *r*) with confidence intervals, effect sizes, degrees of freedom and *P* value noted<br>*Give P values as exact values whenever suitable.*
☒	☐	For Bayesian analysis, information on the choice of priors and Markov chain Monte Carlo settings
☒	☐	For hierarchical and complex designs, identification of the appropriate level for tests and full reporting of outcomes
☒	☐	Estimates of effect sizes (e.g. Cohen's *d*, Pearson's *r*), indicating how they were calculated
☐	☒	Clearly defined error bars<br>*State explicitly what error bars represent (e.g. SD, SE, CI)*

Our web collection on statistics for biologists may be useful.

Software and code

Policy information about availability of computer code

Data collection	*Provide a description of all commercial, open source and custom code used to collect the data in this study, specifying the version used OR state that no software was used.*
Data analysis	Graphpad Prism 7

For manuscripts utilizing custom algorithms or software that are central to the research but not yet described in published literature, software must be made available to editors/reviewers upon request. We strongly encourage code deposition in a community repository (e.g. GitHub). See the Nature Research guidelines for submitting code & software for further information.

Data

Policy information about availability of data

All manuscripts must include a data availability statement. This statement should provide the following information, where applicable:
- Accession codes, unique identifiers, or web links for publicly available datasets
- A list of figures that have associated raw data
- A description of any restrictions on data availability

The coordinates and structure factors have been deposited in the Protein Data Bank under accession codes 6H15 and 6H16. Other data are available from the

corresponding author upon reasonable request. The source data underlying Figures 1b, 1c, 2a, 2b, 2c, 4a, 4b, 4c, 4d, 4e, 4f, 5b, 5d and Supplementary Figure 2 is provided as a Source Data file.

Field-specific reporting

Please select the best fit for your research. If you are not sure, read the appropriate sections before making your selection.

☒ Life sciences ☐ Behavioural & social sciences ☐ Ecological, evolutionary & environmental sciences

For a reference copy of the document with all sections, see nature.com/authors/policies/ReportingSummary-flat.pdf

Life sciences study design

All studies must disclose on these points even when the disclosure is negative.

Sample size	N/A
Data exclusions	A single datapoint was omitted due to a missing value (Figure 4a).
Replication	Yes
Randomization	N/A
Blinding	N/A

Reporting for specific materials, systems and methods

Materials & experimental systems

n/a	Involved in the study	
☒	☐	Unique biological materials
☐	☒	Antibodies
☐	☒	Eukaryotic cell lines
☒	☐	Palaeontology
☒	☐	Animals and other organisms
☒	☐	Human research participants

Methods

n/a	Involved in the study	
☒	☐	ChIP-seq
☒	☐	Flow cytometry
☒	☐	MRI-based neuroimaging

Antibodies

Antibodies used	For Immunoblotting: mouse anti-α-Tubulin (Sigma Aldrich, T5168, 1:10,000); mouse anti-actin (MP Biomedicals (CLONE C4), 08691002, 1:10,000); rabbit anti-LRP6 (Cell Signaling (C5C7), #2560, 1:1,000); rabbit anti-Phospho-LRP6 (Ser1490) (Cell Signaling, #2568, 1:500). All goat secondary antibodies were conjugated with either Alexa Fluorphores (Life Technologies, A-21109, 1:8,000) or IRDyes (LI-COR, P/N 926-32210, 1:8,000). For immunofluorescence: rabbit anti-Ki67 (Abcam, 15580, 1:100), rabbit anti-Lysozyme (Dako, A0099, 1:500), mouse anti-ChgA (Santa Cruz (C-12), sc-393941, 1:200). As secondary antibodies goat anti-rabbit IgG-488 (Life Technologies, A-21441, 1:300) and goat anti-mouse-IgG-568 (Life Technologies, A-11004, 1:300) were used. Hoechst 33342 (Sigma, B-2261) was used at 1:500 and Phalloidin (Ph647) (Cell Signaling, #8940) at 1:50.For Immunoblotting: mouse anti-α-Tubulin (Sigma Aldrich, T5168, 1:10,000); mouse anti-actin (MP Biomedicals (CLONE C4), 08691002, 1:10,000); rabbit anti-LRP6 (Cell Signaling (C5C7), #2560, 1:1,000); rabbit anti-Phospho-LRP6 (Ser1490) (Cell Signaling, #2568, 1:500). All goat secondary antibodies were conjugated with either Alexa Fluorphores (Life Technologies, A-21109, 1:8,000) or IRDyes (LI-COR, P/N 926-32210, 1:8,000). For immunofluorescence: rabbit anti-Ki67 (Abcam, 15580, 1:100), rabbit anti-Lysozyme (Dako, A0099, 1:500), mouse anti-ChgA (Santa Cruz (C-12), sc-393941, 1:200). As secondary antibodies goat anti-rabbit IgG-488 (Life Technologies, A-21441, 1:300) and goat anti-mouse-IgG-568 (Life Technologies, A-11004, 1:300) were used. Hoechst 33342 (Sigma, B-2261) was used at 1:500 and Phalloidin (Ph647) (Cell Signaling, #8940) at 1:50.
Validation	*Describe the validation of each primary antibody for the species and application, noting any validation statements on the manufacturer's website, relevant citations, antibody profiles in online databases, or data provided in the manuscript.*

Eukaryotic cell lines

Policy information about cell lines

Cell line source(s)	Human embryonic kidney (HEK)293T cells (ATCC CRL-3216)
Authentication	N/A
Mycoplasma contamination	All cell lines were tested negative for mycoplasma contamination.
Commonly misidentified lines (See ICLAC register)	N/A
